# CD26 expression on T cell lines increases SDF-1-*α*-mediated invasion

**DOI:** 10.1038/sj.bjc.6605236

**Published:** 2009-08-04

**Authors:** P A Havre, M Abe, Y Urasaki, K Ohnuma, C Morimoto, N H Dang

**Affiliations:** 1 Division of Hematology/Oncology, University of Florida, Gainesville, FL 32610, USA; 2 Department of Hematologic Malignancies, Nevada Cancer Institute, Las Vegas, NV 89135, USA; 3 Department of Clinical Immunology, Institute of Medical Science, University of Tokyo, Tokyo 108-8639, Japan

**Keywords:** CD26, CD45, T cell lines, invasion, MAP kinase, MMP-9

## Abstract

**Background:**

CD26 is a multifunctional membrane-bound glycoprotein that regulates tumour growth in addition to its other activities. Because disease aggressiveness is correlated with CD26 expression in several T-cell malignancies, we decided to investigate the invasiveness of cells expressing different levels of CD26.

**Methods:**

To assess CD26 involvement in cell invasion, we performed *in vitro* invasion assays with human T cell lines expressing different levels of CD26. These included the parental CD26-positive T-lymphoblast cell line HSB-2 and clones infected with a retrovirus expressing siRNA vectors that either targeted CD26 or encoded a missense siRNA, and the parental CD26-negative T-leukaemia cell line Jurkat and clones expressing CD26. CD26 expression in these cell lines was evaluated by flow cytometry and western immunoblotting. CXCR4 expression, phosphorylation of signalling kinases, and MMP-9 secretion were also evaluated by western immunoblotting, whereas MMP-9 activity and the effect of kinase and CD45 inhibitors on activity were measured by zymography of conditioned media.

**Results:**

The presence of CD26 enhanced stromal-cell-derived factor-1-*α* (SDF-1-*α*)-mediated invasion of T cell lines. This process was regulated in part by the PI-3K and MEK1 pathways, as indicated by increased phosphorylation of p44/42 MAP kinase and Akt in the presence of SDF-1-*α* and the effect of their respective inhibitors on MMP-9 secretion and *in vitro* invasion. In addition, CD26-associated enhancement of SDF-1-*α*-induced invasion was decreased when CD45 was inhibited.

**Conclusions:**

Our results indicate that the expression of CD26 in T cell lines leads to increased SDF-1-*α*-mediated invasion in an *in vitro* system and that this is controlled in part by the PI-3K and MEK1 pathways. The data also suggest that CD26 enhancement of invasion may be mediated by CD45, however, more studies are required to confirm this involvement.

CD26 (DPPIV) is a multifunctional membrane-bound glycoprotein present on the surface of many cell types. It has three domains: an extracellular domain which contains its enzyme activity, a transmembrane region, and a short cytoplasmic tail of six amino acids, enabling it to interact with both intra- and extracellular molecules. CD26 is critical in T-cell biology, as a marker of T-cell activation and by virtue of its involvement in various signalling pathways (Fox *et al*, 1984; Dang *et al*, 1990a, 1990b; Torimoto *et al*, 1991). Association with various proteins including fibroblast-activation protein-*α* (FAP*α*), plasminogen, adenosine deaminase, CD45, collagen, and fibronectin, influences its activity. As a result, CD26 has an important, but complex, function in tumour behaviour, with its biological effect dependent on the tumour type and the microenvironment. Likely as a result of this multifunctional character, CD26 is associated with a high level of clinical aggressiveness in some tumours but a lower level in others (Iwata and Morimoto, 1999; Kajiyama *et al*, 2002; Havre *et al*, 2008). For example, it is a marker of aggressive disease for certain subsets of T-cell non-Hodgkin's lymphomas/leukaemias, with expression of CD26 on T-lymphoblastic lymphomas/acute lymphoblastic leukaemia cells being associated with a worse outcome compared with CD26-negative tumours (Carbone *et al*, 1995). CD26 is also expressed at high levels on malignant mesothelioma (Inamoto *et al*, 2007) and renal carcinoma cells (Droz *et al*, 1990; Stange *et al*, 2000; Inamoto *et al*, 2006). Two recent studies also correlate CD26 expression and tumourigenesis. In an immunochemical analysis of 152 patients with gastric gastrointestinal stromal tumours, CD26 was found to be associated with a poorer overall survival (Yamaguchi *et al*, 2008). In addition, CD26 can serve as a prognostic marker in B-cell chronic lymphocytic leukaemia (Cro *et al*, 2009).

Stromal-cell-derived factor-1-*α* (SDF-1-*α*)/CXCL12 and its receptor CXCR4 have been shown to be critical in tumourigenesis. Stromal-cell-derived factor-1-*α* is constitutively expressed in most tissues (Shirozu *et al*, 1995), whereas CXCR4 is expressed in many cancer cell lines and tissues (Burger *et al*, 1999; Muller *et al*, 2001; Bertolini *et al*, 2002; Schrader *et al*, 2002) and is able to mediate the metastasis of a number of cancers (Zlotnik, 2006).

Previous studies from our group and others showed that CD26 is able to mediate extracellular matrix binding (Cheng *et al*, 1998, 2003; Sato *et al*, 2005) and tumourigenesis (Sato *et al*, 2005). To evaluate the mechanism associated with CD26 involvement in cell invasion, we used two human T cell lines differing in CD26 expression level. The first is the parental CD26-positive T-lymphoblast cell line HSB-2 and clones infected with retrovirus expressing siRNA vectors that either targeted CD26 or encoded a missense siRNA. The second is the parental CD26-negative T-leukaemia cell line Jurkat and clones transfected with an empty vector or with a vector encoding CD26. *In vitro* invasion assays were performed in the presence or absence of SDF-1-*α*. Our results showed that expression of CD26 enhanced SDF-1-*α*-mediated invasion of human T cell lines. In addition, treatment with the protein kinase inhibitors LY294002 and PD98059 significantly inhibited MMP-9 secretion and cellular invasion. These data hence indicate that p44/42 MAP kinase (Erk 1/2) and the downstream mediator of the PI-3K signalling pathway, Akt, are important regulators of *in vitro* SDF-1-*α*-induced invasion. In addition, inhibition of CD45 activity reduced both MMP-9 secretion and cellular invasion, suggesting that CD26 may exert its effect on invasion through its interaction with CXCR4 and CD45.

## Materials and methods

### Reagents

Bovine serum albumin (BSA), sodium vanadate, *β*-glycerophosphate, sodium fluoride, polybrene (hexadimethrine bromide), puromycin, TX-100, and gelatin type A (porcine) were purchased from Sigma-Aldrich (St Louis, MO, USA). LY294002 and SB203580 were from Promega (Madison, WI, USA) whereas PD98059 and the PTPase CD45 inhibitor, *N*-(9,10-dioxo-9,10-dihydro-phenanthrene-2-yl)-2,2-dimethyl-propionamide, were from Calbiochem (EMD Biosciences Inc., San Diego, CA, USA).

### Cell culture

Jurkat cells were originally obtained from the American Type Culture Collection (ATCC, Manassas, VA, USA) and maintained in RPMI 1640 (Hyclone, Logan, UT, USA). Jurkat cells transfected with CD26 have been described previously (Tanaka *et al*, 1992). HSB-2 cells were also obtained from ATCC and maintained in Iscove's modified Dulbecco's medium (ATCC). CD26-depleted clones were generated using siRNA as described previously (Yamochi *et al*, 2005). All cell media contained 10% fetal bovine serum (Hyclone), penicillin (100 u ml^−1^), and streptomycin (100 *μ*g ml^−1^).

### siRNA

CD26 sense and anti-sense oligonucleotides were hybridized and inserted into the RNAi-Ready pSIREN-RetroQ vector (Clontech, Mountain View, CA, USA) (Yamochi *et al*, 2005). The target sequence was 1768–1786 downstream from the start codon of CD26 mRNA (5′-GATCCGATCATGCATGCAATCAACTTCAAGAGAGTTGATTGCATGCATGATCTTTTTTGGAAG-3′). A missense siRNA vector was also generated and has been described (5′-GATCCGATCTTGCAAGCAAACAACTTCAAGAGAGTTGTTTGCTTGCAAGATCTTTTTTGGAAG-3′) (Yamochi *et al*, 2005). GP2-293 cells were transfected with siRNA vectors and VSV-G (Clontech) using Fugene 6 transfection reagent (Roche Diagnostics, Indianapolis, IN, USA). The virus supernatants were collected 48 h following transfection and frozen until used. HSB-2 cells were infected with viral supernatants in the presence of polybrene (4 *μ*g ml^−1^) and selected with 2.5 *μ*g ml^−1^ puromycin. Clones were obtained by limiting dilution.

### Flow cytometry

Cells were washed once with staining buffer (PBS containing 0.5 mM EDTA and 1% BSA) and incubated on ice for 30 min with phycoerythrin-conjugated mouse anti-CD26 or isotypic mouse IgG (Beckman Coulter, Fullerton, CA, USA). After washing with staining buffer twice, the cells were re-suspended in PBS. The surface expression of CD26 was analysed by Reflection (iCyt Visionary Bioscience, Champaign, IL, USA) using WinList software (Verity Software House, Topsham, ME, USA).

### Invasion assay

T-cell invasion assays were carried out using 24-well plates containing 12 transwell inserts with 5.0 *μ*m pores (Corning Inc., Corning, NY, USA) as previously described (Shaw, 2005; Redondo-Munoz *et al*, 2006). Wells were coated with ECL Cell Attachment Matrix (Upstate, Temecula, CA, USA) and allowed to dry before reconstitution in serum-free media (SFM). When SDF-1-*α* (R&D Systems Inc., Minneapolis, MN, USA) was added to the media below transwells, it was used at 20 nM. Cells were washed in SFM, then resuspended in SFM containing 0.1% BSA. Cells (2.5 × 10^5^) were added to transwells and also to wells without membranes to obtain total cell number. After 24 h, the transwells were rinsed with PBS above the membrane, fixed in methanol, stained with 0.2% cresyl violet, and rinsed in water. Invasion level was determined by dividing the number of cells that passed through the coated transwell by the total cell number (determined using a hemocytometer or Coulter counter). When inhibitors were present, cells were preincubated with the inhibitor for 60 min at 37°C before adding cells to transwells. Total cell number for this set was determined using cells incubated with the inhibitor.

### Cell lysates

Cells were lysed using RIPA buffer (20 mM Tris-Cl (pH 7.5), 140 mM NaCl, 1% NP-40, 0.5% deoxycholate, 0.1% sodium dodecyl sulphate(SDS))containing Halt protease inhibitor cocktail with 5 mM EDTA (Pierce, Rockford, IL, USA) and phosphatase inhibitors (5 mM NaF, 1 mM *β*-glycerophosphate, and 1 mM sodium vanadate). Protein concentration was determined using the bicinchoninic acid protein assay reagent (Pierce).

### Induction of phosphorylation

To detect SDF-1-*α*-induced phosphorylation, 2 × 10^6^ cells per well were grown overnight in a six-well plate (previously coated with 1% BSA–PBS) in SFM containing 0.1% BSA. Stromal-cell-derived factor-1-*α* was added to cells at a final concentration of 10 nM for 0–20 min before dilution in cold PBS containing 1 mM sodium vanadate and cells were harvested.

### Western blots

Equal amounts of protein were run on 7.5 or 10% polyacrylamide gels. For detection of CD26, samples were heated at 37°C instead of 100°C in Laemmli sample buffer because high temperatures destroyed the epitope recognised by the antibody. Following transfer, blots were blocked, then probed with one of the following antibodies: CD26 (R&D Systems Inc.); CXCR4 (Millipore, Billerica, MA, USA); Akt and phospho-Akt (Ser473); p44/42 MAPK (Erk 1/2); and phospho-p44/42 MAPK (Cell Signaling, Beverly, MA, USA); and MMP-9 (R&D Systems Inc.). Horseradish-peroxidase-conjugated secondary antibodies and the detection reagent, SuperSignal West Dura Extended Duration Substrate, were from Pierce. Blots were scanned using a Kodak Image Station 2000R or 4000R (New Haven, CT, USA). Alternatively, Li-Cor IRDye-conjugated secondary antibodies were used and blots were scanned using an Odyssey imager (Li-Cor Biosciences, Lincoln, NE, USA).

### Zymography

Six- or twelve-well plates were coated overnight with 1% BSA–PBS. Cells (2 × 10^6^) were suspended in SFM containing 0.1% BSA and incubated for 24 h before the addition of SDF-1-*α* (10 nM). After 24 h, cells were pelleted and the conditioned media was combined with Laemmli sample buffer without reducing agent and run on a 7.5% SDS-polyacrylamide gel containing 1 mg ml^−1^ gelatin as previously described (Gum *et al*, 1997). The gel was then incubated at room temperature in 2.5% TX-100 (w/v) for 2 h, then overnight at 37°C in 20 mM Tris-Cl (pH 7.4), 136 mM NaCl, 10 mM CaCl_2_. Bands were visualised as clear zones following staining with Coomassie blue (Leber and Balkwill, 1997). Gels were scanned using a Kodak Image Station 4000R.

### Statistical analysis

Wherever applicable, statistical significant differences were evaluated by determining the standard error of the mean.

## Results

### Expression of CD26 on HSB-2 cell lines

There is substantial evidence to support the involvement for CD26- and DPPIV-related proteins in migration and/or invasion (Ghersi *et al*, 2001, 2006; Ikushima *et al*, 2002; Chen, 2003; Gonzalez-Gronow *et al*, 2005; Yu *et al*, 2006). Although the mechanisms may differ depending on the cell type, several chemokines we initially evaluated had no effect on cell invasion, whereas SDF-1-*α* did, which was somewhat unexpected based on earlier studies (Shioda *et al*, 1998; Christopherson *et al*, 2002). To determine whether CD26 was involved in SDF-1-*α*-mediated cell invasion, we infected parental HSB-2 cells either with an siRNA that targeted CD26 to create CD26-depleted clones or with a missense siRNA to serve as a control. Following selection with puromycin and cloning by limiting dilution, several CD26-depleted clones were selected for further studies.

**Figure 1:**
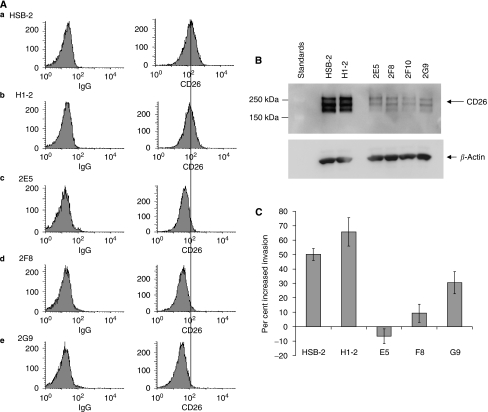
SDF-1-*α*-mediated invasion is higher in HSB-2 cells than in CD26-depleted clones. (**A**) Cell-surface expression of CD26 on HSB-2 cell lines. (**a**) HSB-2 parental (**b**) H1-2 (**c**) 2E5 (**d**) 2F8 (**e**) 2G9. Left: cells incubated with PE-labeled isotypic control IgG. Right: cells incubated with PE-labeled anti-CD26 antibody. (**B**) Expression of CD26 in whole-cell extracts. Equal amounts of protein were run in each lane of a 7.5% gel, transferred to nitrocellulose, and probed with anti-CD26 antibody. Lanes contained HSB-2 parental cells, H1-2, and four clones depleted of CD26: 2E5, 2F8, 2F10, and 2G9. Note that CD26 runs as a dimer (see Materials and Methods). The multiple bands are due to differences in glycosylation. (**C**) Invasion assay (details in Materials and Methods). Assays were carried out for 24 h, at which time the cells were counted in all wells. Percentage invasion is the number of cells which passed through the transwell divided by the total cell number. Error bars are shown for the standard error of the mean.

HSB-2 cell lines were evaluated for CD26 surface expression by flow cytometry as described in Materials and methods. HSB-2 cells and cells infected with missense siRNA, H1-2, expressed CD26 (Figure 1A). In contrast, CD26 expression was undetectable for the three clones 2E5, 2F8, and 2G9. To confirm CD26 expression, we ran whole-cell lysates on SDS gels, transferred to nitrocellulose, and probed with an anti-CD26 antibody. Both HSB-2 and H1-2 expressed CD26, but four CD26-depleted clones had a much lower level of CD26 than either the parental HSB-2 or H1-2 cells (Figure 1B). Note that because extracts were heated at 37°C instead of 100°C before loading, the detected size of CD26 was approximately 220 kDa instead of 110 kDa (Ghersi *et al*, 2002).

### SDF-1-*α*-mediated invasion is higher in HSB-2 cells than in CD26-depleted clones

To evaluate the function of CD26 in invasion, SDF-1-*α*, was added underneath the transwells to obtain a final concentration of 20 nM, as described in Materials and Methods. Following incubation for 24 h, cells were counted for each well. No cells remained attached to the transwell support as confirmed by cresyl violet staining (data not shown). The presence of SDF-1-*α* resulted in increased invasion for both the HSB-2 parent cell line and H1-2 expressing missense siRNA. However, invasion was not increased for 2E5, was marginally increased for 2F8, and moderately increased for 2G9 (Figure 1C). Of note is that the difference in invasive activity of the three CD26-depleted clones was statistically significant when compared to the CD26-expressing cells. Results are expressed as percent increased invasion because of variation in the absolute values for experiments run on different days.

### Expression of CD26 on Jurkat cell lines

**Figure 2:**
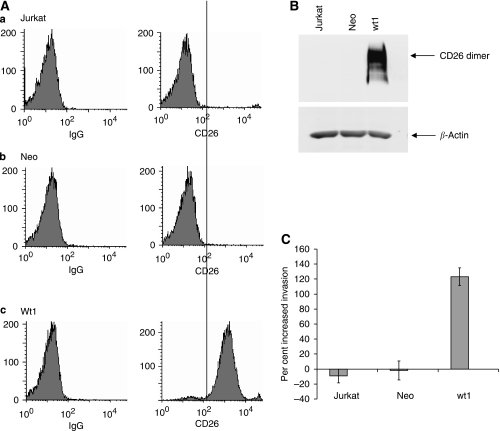
SDF-1-*α*-mediated invasion is elevated in Jurkat transfectants expressing CD26. (**A**) Cell-surface expression of CD26 on Jurkat cell lines. (**a**) Jurkat-parental, CD26-negative (**b**) Jurkat-Neo (**c**) Jurkat-Wt1. Left: cells incubated with PE-labeled isotypic control IgG. Right: cells incubated with PE-labeled anti-CD26 antibody. (**B**) Expression of CD26 in whole-cell extracts. Equal amounts of protein were run in each lane of a 7.5% gel, transferred to nitrocellulose, and probed with anti-CD26 antibody. Lanes contained Jurkat-parental, Neo cells transfected with empty vector, and wt1 transfectant expressing high levels of CD26, respectively. Note that CD26 runs as a dimer. (**C**) Invasion assay (as described for Figure 1C). Error bars are shown for the standard error of the mean.

Another cell type was used to further evaluate CD26 involvement in SDF-1-*α*-mediated invasion. Surface expression of CD26 was determined as previously described for HSB-2 cells. CD26 was not detected on the surface of parental Jurkat cells or Jurkat cells transfected with the empty vector Neo (Figure 2A). In contrast, CD26 was highly expressed on the cell surface of the Jurkat transfectant wt1. Western blotting with anti-CD26 antibody confirmed the results from flow cytometry (Figure 2B). There was no CD26 protein detected in either the parental Jurkat cell line or Jurkat cells transfected with the empty vector Neo. However, Jurkat cells transfected with wild-type CD26 expressed a high level (wt1) of CD26.

### SDF-1-*α*-mediated invasion is elevated in Jurkat transfectants expressing CD26

Others have shown that CD26-negative Jurkat cells are able to migrate in response to SDF-1-*α* (Chernock *et al*, 2001; Tan *et al*, 2006), and that migration was dependant on CXCR4 (Tan *et al*, 2006). Here, in a comparison of parental Jurkat cells, cells containing the empty vector, and transfectants expressing high levels of CD26, the CD26-expressing cells exhibited much higher invasive activity in response to SDF-1-*α* as compared to the other cells (Figure 2C). The addition of SDF-1-*α* did not lead to increased invasive activity for the parental Jurkat cells or for Jurkat cells transfected with the empty vector.

### CXCR4 level is not dependant on CD26 expression and is not affected by SDF-1-*α*

**Figure 3:**
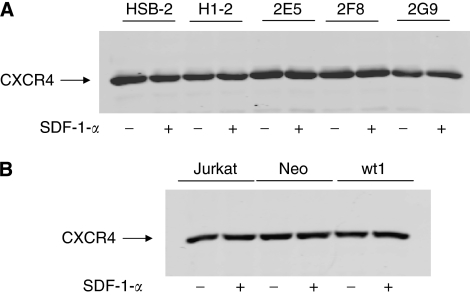
CXCR4 level is not dependant on CD26 expression and is not affected by SDF-1-*α*. Expression of CXCR4 in whole-cell lysates. Equal amounts of protein were run in each lane of a 7.5% gel, transferred to nitrocellulose, and probed with anti-CXCR4 antibody. (**A**) Lanes contained HSB-2 parental cells, H1-2 (parental cells with control siRNA), and three CD26-depleted clones: 2E5, 2F8, and 2G9. (**B**) Lanes contained CD26-negative Jurkat parental cells, Neo (parental cells with empty vector), and wt1, a CD26-overexpressing clone. When SDF-1-*α* was present (+), it was at a final concentration of 10 nM.

Stromal-cell-derived factor-1-*α* presence has been shown to upregulate CXCR4 expression in the prostate cancer cell line PC-3 (Kukreja *et al*, 2005). We therefore considered the possibility that there might be a difference in the expression of CXCR4 among the parental cells and clones differing in CD26 levels. Furthermore, the possibility that CD26-expressing cells might upregulate CXCR4 in response to SDF-1-*α*, whereas CD26-negative cells might be resistant to change was also evaluated. Cells were maintained overnight in SFM containing 0.1% BSA, and SDF-1-*α* was added the following day at a final concentration of 10 nM. Whole-cell lysates were prepared and run on gels, transferred to nitrocellulose, and probed with anti-CXCR4 antibody. Our data showed that CXCR4 level was not affected by SDF-1-*α* (Figure 3). In addition, CXCR4 expression did not vary significantly among parental cells expressing CD26 (HSB-2 and H1-2) and CD26-depleted clones (2E5, 2F8, and 2G9) or among CD26-negative parental Jurkat cells (Jurkat and Neo) and a CD26-overexpressing clone (wt1).

### SDF-1-*α* induces phosphorylation of signalling proteins in T cell lines

**Figure 4:**
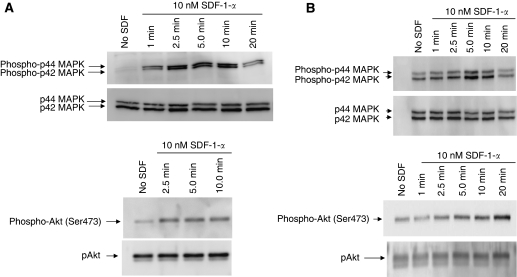
SDF-1-*α* induces phosphorylation of signalling proteins in T cell lines. (**A**) Activation of signalling kinases in HSB-2 cells following incubation with SDF-1-*α*. Cells were incubated with 10 nM SDF-1-*α* for the indicated times, run on gels, transferred to nitrocellulose, then probed with site-specific antibodies. Top left, phosphorylated MAPK p44/42 (thr202/tyr204); bottom left, phosphorylated Akt (Ser473). Data depicted are representative of two separate experiments. (**B**) Activation of signalling kinases in Jurkat wt1. Top right, phosphorylated MAPK p44/42 (thr202/tyr204); bottom right, phosphorylated Akt (Ser473). Data depicted are representative of three separate experiments. Total p44/42 MAPK and Akt are shown below their respective phosphorylated kinases.

Phosphorylation of several signalling proteins has been shown to be important in *in vitro* cell migration or invasion for various cell types. For example, Akt activation is required for migration of HeLa cells (Peng *et al*, 2005), whereas activation of PI-3K and p44/42 MAPK is required for SDF-1-*α*-dependent T-cell migration (Sotsios *et al*, 1999). In prostate cancer cells, PI-3 kinase and p44/p42 MAPK were shown to be important for SDF-1-*α*-mediated invasion (Chinni *et al*, 2006), whereas in breast cancer cells PI-3K, but not p44/p42 MAPK, was required (Fernandis *et al*, 2004). To determine the signalling pathways involved in CD26-regulated invasive activity of T cell lines, we evaluated the phosphorylation status of the key intracellular signalling molecules p44/42 MAPK and Akt following incubation of cells with SDF-1-*α* at various time points, as described in Materials and methods. For HSB-2 cells, there was a minimal level of p44/42 MAPK (Erk 1/2) phosphorylation in the absence of SDF-1-*α* (Figure 4A). Following the addition of SDF-1-*α*, phosphorylation level increased between 1 and 10 min of incubation, but decreased substantially after 20 min of incubation. Phosphorylation of Akt was also low in the absence of SDF-1-*α*, but increased by 2.5 min on incubation with the chemokine. Stromal-cell-derived factor-1-*α*-mediated phosphorylation of key signalling molecules was similarly evaluated for the CD26-expressing Jurkat clone wt1 (Figure 4B). Phosphorylation of p44/42 MAPK was induced following incubation with SDF-1-*α*, peaking by 5 min and returning to baseline level by 20 min. In addition, phosphorylation of Akt was induced by SDF-1-*α*, gradually increasing in intensity between 0 and 20 min of incubation.

### Kinase inhibitors decrease invasion in T cell lines

To confirm the function of key signalling pathways in SDF-1-*α*-mediated invasion of CD26-expressing T cell lines, we incubated cells with specific kinase inhibitors before being evaluated in *in vitro* invasion assays (Table 1). Inhibition of PI-3K by LY294002 and MEK1 by PD98059 resulted in a significant decrease in invasive activity for both cell types. In contrast, the p38 MAPK inhibitor, SB203580, had a marginal effect on invasion. Because Akt is a downstream effector of PI-3K, these results indicate that activation of both Akt and p44/42 MAPK, but not p38 MAPK, is required for SDF-1-*α*-dependent invasion of CD26-expressing T cell lines.

### MMP-9 is induced in HSB-2 cells but not in CD26-depleted clones

**Figure 5:**
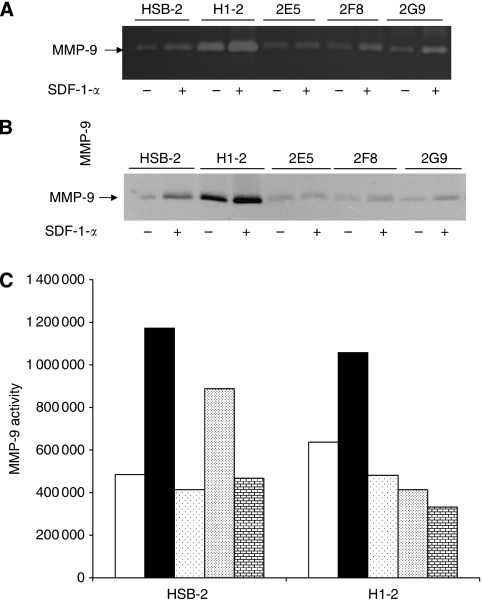
MMP-9 is induced in HSB-2 cells but not in CD26-depleted clones. Cells were incubated with or without SDF-1-*α* (see Materials and methods). Cells were harvested at 24 h and the conditioned media was run on gels. (**A**) Equal volumes of conditioned media were loaded on an acrylamide gel containing 0.1% gelatin. Following staining, clear zones corresponding to digested gelatin could be detected. (**B**) Conditioned media was run on a 7.5% acrylamide gel and then probed with anti-MMP-9 antibody following transfer to nitrocellulose. (**C**) Inhibitors were added 30 min before the addition of SDF-1-*α* to both HSB-2 and H1-2 cells. The PI-3K inhibitor, LY294002, was added at a concentration of 12 *μ*M, the MEK1 inhibitor, PD98059, was at 11 *μ*M, and the CD45 inhibitor, *N*-(9,10-dioxo-9,10-dihydro-phenanthrene-2-yl)-2,2-dimethyl-propionamide, was at 1.5 *μ*M. No SDF (clear bars), SDF and vehicle (solid bars), SDF and MEK1 inhibitor (dots, low density), SDF and CD45 inhibitor (dots, high density), SDF and PI-3K inhibitor (bricks). Gels were scanned and the clear zones quantified after inverting the image. Data depicted are representative of three experiments.

Because previous studies have reported a link between MMP-9 production and invasion (Kim *et al*, 2001; Redondo-Munoz *et al*, 2006), we examined potential MMP-9 induction following a 24 h treatment with SDF-1-*α*. Conditioned media was analysed by zymography and by western blots. For zymography, clear zones represent digested gelatin present in the gel. MMP-9 was induced in the CD26-expressing parental HSB-2 cells and H1-2 when SDF-1-*α* was present. In contrast, MMP-9 was not induced in the CD26-depleted clone 2E5, but moderately induced in clones 2F8 and 2G9 as shown by the clear zones (Figure 5A). This pattern parallels the one obtained for invasion where 2E5 showed no increase in invasion in the presence of SDF-1-*α*, whereas the other two clones, 2F8 and 2G9, showed a moderate increase in invasion (Figure 1A). Western blotting of the conditioned media further confirmed that MMP-9 secretion increased in HSB-2 and H1-2 cells, but did not increase for 2E5 and moderately increased for 2F8 and 2G9 (Figure 5B).

Because Akt has been shown previously to regulate MMP-9 induction (Kim *et al*, 2001), we next examined the potential function of Akt in our system by pre-incubating HSB-2 or H1-2 cells with the PI-3K inhibitor LY294002 for 30 min before the addition of SDF-1-*α*. MMP-9 secretion was decreased in the presence of the inhibitor, indicating that Akt is involved in SDF-1-*α*-mediated secretion of MMP-9 in CD26-expressing cells (Figure 5C). In addition, inhibition of p44/42 MAPK activation using the MEK1 inhibitor PD98059 and CD45 activity using *N*-(9,10-dioxo-9,10-dihydro-phenanthrene-2-yl)-2,2-dimethyl-propionamide also reduced MMP-9 secretion in both HSB-2 and H1-2 cells (Figure 5C).

### Invasion is inhibited in the presence of a CD45 inhibitor

**Figure 6:**
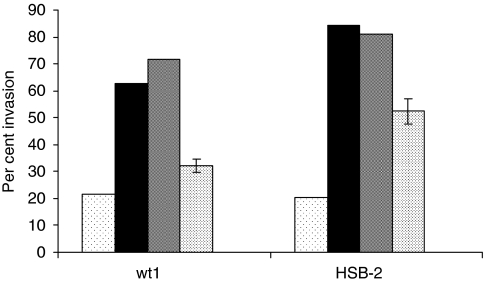
CD45 inhibition decreases invasion in T cell lines. Cells (Jurkat wt1 or HSB-2) were incubated with (solid bars) or without SDF-1-*α* (dots, low density), DMSO and SDF-1-*α* (grey) or with SDF-1-*α*, DMSO, and 6 *μ*M CD45 inhibitor (dots, high density) for 1 h before start of invasion assay. Invasive activity was determined as described previously. The standard error is shown for cells incubated with the inhibitor, because three wells each were used for this group.

Because several studies have shown previously that CD45 expression enhances migration (Harvath *et al*, 1991; Fernandis *et al*, 2003; Okabe *et al*, 2006), and because our group has demonstrated that CD26 association with CD45 results in enhanced phosphorylation of several signalling kinases, including p44/42 MAPK (Torimoto *et al*, 1991; Hegen *et al*, 1997; Ishii *et al*, 2001), we were interested in the effect of CD45 inhibition on SDF-1-*α*-mediated invasion in CD26 expressing cells. T cells were incubated with a CD45 inhibitor for 1 h before performing the invasion assays. CD45 inhibitor decreased invasive activity for both CD26-expressing parental HSB-2 cells and Jurkat wt1 transfectants (Figure 6). The level of invasive activity in the presence or absence of SDF-1-*α* was consistent with data obtained from previous assays (Figures 1C and 2C). Pre-treatment with the CD45 inhibitor, however, resulted in decreased invasive activity for both cell lines. These results suggest that CD26 may be exerting its effect on SDF-1-*α*-mediated invasion through its association with CXCR4 and CD45.

## Discussion

Because disease aggressiveness is correlated with CD26 expression in several T-cell malignancies (Havre *et al*, 2008), we decided to investigate *in vitro* invasiveness of T cells expressing different levels of CD26. Chemotaxis and invasion of tumour cells are critical for the development of metastasis, which is governed in part by interactions between chemokine receptors on cancer cells and matching chemokines on target organs. Although it is well established that SDF-1-*α* increases migration of cells expressing CXCR4 (Ganju *et al*, 1998; Sotsios *et al*, 1999), only a few studies have demonstrated the effect of SDF-1-*α* on invasion (Fernandis *et al*, 2004; Chinni *et al*, 2006). In prostate cancer cells, PI-3 kinase and p44/p42 MAPK were shown to be important for SDF-1-*α*-mediated invasion (Chinni *et al*, 2006), whereas in breast cancer cells PI-3K, but not p44/p42 MAPK, was required (Fernandis *et al*, 2004).

In addition to protein kinases, several tyrosine phosphatases have been shown to be important for migration. The expression of SHP-1 appears to be inversely correlated with migration in haematopoetic cells from mice (Kim *et al*, 1999), whereas SHP2 overexpression increases SDF-1-*α*-induced chemotaxis in human lymphocytes (Chernock *et al*, 2001). Another tyrosine phosphatase, CD45, has also been shown to enhance cell migration in response to SDF-1-*α* (Harvath *et al*, 1991; Fernandis *et al*, 2003; Okabe *et al*, 2006). However, the effect of CD45 on invasion has not previously been reported. In our studies, invasion was decreased in the presence of a CD45 inhibitor. Association of CD26 with CD45 has been reported to result in increased signal transduction, including increased p44/42 MAPK activity (Torimoto *et al*, 1991; Hegen *et al*, 1997; Ishii *et al*, 2001). Moreover, both CD26 and CD45 associate with CXCR4 in lymphocytes (Herrera *et al*, 2001; Fernandis *et al*, 2003). The consequences of the association of CXCR4 and CD26 are not understood. Binding of SDF-1-*α* triggers internalization of both CXCR4 and CD26 in the T cell line Jurkat, the B-cell line SKW6.4, and peripheral blood lymphocytes (Herrera *et al*, 2001), but the importance of internalization for signalling enhancement is controversial (Neel *et al*, 2005).

CD26 also interacts with several other proteins that could promote its role in invasion. It binds to plasminogen 2*ε* in the prostate cancer cell line 1-LN, triggering an intracellular [Ca^2+^] flux leading to the upregulation of MMP-9 (Gonzalez-Gronow *et al*, 2001). CD26 has also been shown to form heterodimers with FAP*α*, colocalizing at pseudopodia and causing secretion/activation of MMPs in migratory fibroblasts and endothelial cells (Ghersi *et al*, 2002, 2006).

It is well documented that CD26 can cleave and inactivate SDF-1-*α*. It has been shown that SDF-1-*α* is cleaved by soluble CD26 *in vitro* (Ohtsuki *et al*, 1998; Proost *et al*, 1998; Lambeir *et al*, 2001), resulting in the inactivation of its chemotactic activity. In addition, there are several reports of reduced migration in the presence of SDF-1-*α* when cells express CD26 (Shioda *et al*, 1998; Christopherson *et al*, 2002). However, substrate recognition and cleavage efficiency is likely to be dependant on CD26-associated proteins and the local concentration of SDF-1-*α*. Studies using purified CD26 showed *K*_m_ values ranging from 5 to 60 *μ*M for natural Xaa-pro and Xaa-ala peptides with 7–42 residues. However, *in vivo* regulatory peptides act in the pico- and nanomolar range, so micromolar concentrations are not reached (Mentlein, 1999). Therefore, a number of factors including the microenvironment are likely to determine whether CD26 enhances or diminishes invasion.

There is a growing list of malignancies in which CD26 is involved. It is a marker of aggressive disease for selected subsets of T-cell non-Hodgkin's lymphomas/leukaemias (Carbone *et al*, 1995). It is also expressed at high levels on malignant mesothelioma (Inamoto *et al*, 2007) and renal carcinoma cells (Droz *et al*, 1990; Stange *et al*, 2000; Inamoto *et al*, 2006) and was found to be associated with poorer overall survival in patients with gastric gastrointestinal stromal tumours (Yamaguchi *et al*, 2008). Our present work shows that SDF-1-*α*-mediated invasion was enhanced in CD26-expressing T cell lines. Invasion was partially regulated by the PI-3K and MEK1 pathways as shown by increased phosphorylation of p44/42 MAPK and Akt in the presence of SDF-1-*α* and by decreased MMP-9 secretion and *in vitro* invasion in the presence of their respective inhibitors. Our data also suggest that CD26 enhancement of invasion may be mediated by CD45.

## Figures and Tables

**Table 1:** Invasion is inhibited in the presence of kinase inhibitors

**Inhibitor**	**Inhibition for wt1 (%)**	**Inhibition for H1-2 (%)**
LY294002	70.4	48.5
PD98059	50.0	35.4
SB203580	12.8	23.8

Cells were incubated with inhibitors for 1 h before the start of invasion assays. The phosphatidylinositol 3-kinase (PI-3K) inhibitor, LY294002, and the MEK1 inhibitor, PD98059, were used at a concentration of 50 *μ*M, whereas the p38 MAP kinase inhibitor, SB203580, was used at a concentration of 10 *μ*M. Invasion level was determined following 24 h of incubation in the presence of SDF-1-*α*. Per cent inhibition=per cent invasion in the absence of inhibitor−per cent invasion in the presence of inhibitor/per cent invasion in the absence of inhibitor. Data depicted are representative of two separate experiments.
